# Educational video for adherence to vaginal pessary in pelvic organ prolapse treatment

**DOI:** 10.1590/0034-7167-2023-0515

**Published:** 2024-09-30

**Authors:** Karine de Castro Bezerra, Maria Luziene de Sousa Gomes, Roger Rodrigues da Silva, Dayana Maia Saboia, José Ananias Vasconcelos, Camila Moreira Teixeira Vasconcelos, Mônica Oliveira Batista Oriá

**Affiliations:** I Universidade Federal do Ceará. Fortaleza, Ceará, Brazil.; II Universidade Federal do Maranhão. São Luís, Maranhão, Brazil.; III Universidade Federal do Ceará, Complexo Hospitalar, Empresa Brazileira de Serviços Hospitalares. Fortaleza, Ceará, Brazil.

**Keywords:** Health Communication, Instructional Film and Video, Pelvic Organ Prolapse, Pessaries, Treatment Adherence and Compliance, Comunicación en Salud, Película y Video Educativos, Prolapso de Órgano Pélvico, Pesarios, Cumplimiento y Adherencia al Tratamiento

## Abstract

**Objectives:**

to construct and assess an educational video to promote the adherence of women with pelvic organ prolapse to vaginal pessary use.

**Methods:**

this is a methodological study, with a longitudinal design and quantitative analysis. The pre-production (synopsis, argument, script, storyboard), production and post-production stages were covered. Content and technical assessments were carried out by judges from the health and communication areas, respectively, and appearance assessment by the target audience.

**Results:**

the video was the first to be developed on the topic on the national scene, considered assessed from the point of view of appearance and content, presenting an overall Content Validity Index of 0.99 and a level of agreement among judges of 91.1% to 100%. Assessment by the target audience reached a percentage of 96% to 100%.

**Conclusions:**

the educational video is an instrument capable of promoting adherence to pessary in women indicated for this therapeutic approach.

## RESUMEN

**Objetivos:**

construir y evaluar un video educativo para promover la adherencia de mujeres con prolapso de órganos pélvicos al uso de pesarios vaginales.

**Métodos:**

se trata de un estudio metodológico, con diseño longitudinal y análisis cuantitativo. Se cubrieron las etapas de preproducción (sinopsis, argumento, guión, storyboard), producción y postproducción. Las evaluaciones de contenido y técnica fueron realizadas por jueces de las áreas de salud y comunicación, respectivamente, y la evaluación de apariencia por parte del público objetivo.

**Resultados:**

el video fue el primero desarrollado sobre el tema en el panorama nacional, considerado evaluado desde el punto de vista de apariencia y contenido, presentando un Índice de Validez de Contenido global de 0,99 y un nivel de acuerdo entre jueces de 91,1% a 100%. La evaluación por parte del público objetivo alcanzó un porcentaje del 96% al 100%.

**Conclusiones:**

el video educativo es un instrumento capaz de promover la adherencia al pesario en mujeres indicadas para este abordaje terapéutico.

## INTRODUCTION

Pelvic organ prolapse (POP) is the downward uterus displacement and/or vaginal wall compartments and its associated organs (bladder, rectum and intestine)^(1)^. POP has implications in several areas of quality of life, contributing to changes in physical health conditions, cognitive functions, sexual satisfaction, daily activities, emotional well-being, family and social life, causing sexual problems, social isolation, low self-esteem and depression^(2, 3)^. The prevalence of POP varies greatly between studies, ranging from approximately 3% to 50%^(4)^. Women with POP present a variety of urinary, intestinal and sexual symptoms that can significantly compromise their psychological and emotional well-being as well as their quality of life^(5)^.

Treatment options for POP include conservative measures such as pelvic floor muscle exercises and the use of a vaginal pessary, in addition to more interventional measures such as surgery^(6, 7)^. A vaginal pessary is a device inserted into the vagina to provide structural support to one or more of the descending vaginal compartments. There is a wide variety of pessaries available on the market. They are intravaginal devices, usually made of silicone, that support the pelvic organs and place them in the anatomically correct position. Furthermore, it allows symptomatic control with the added benefit of postponing or avoiding surgical intervention^(1, 8)^.

Despite the many benefits that a pessary offers, the choice and continuity of this treatment for women is complex and involves areas related to knowledge, motivation and confidence^(9)^. Educational interventions can maximize the acceptance of women with POP to use a pessary, in addition to improving healthcare in relation to adherence and monitoring of patients who use the device.

From this perspective, this topic falls within goals 3 (ensuring a healthy life and promote well-being for all at all ages) and 5 (achieving gender equality and empower all women and girls), listed among the 2030 Agenda for the United Nations (UN) Sustainable Development Goals (SDG)^(10)^.

An educational intervention method to improve literacy on POP conservative management using vaginal pessaries is digital means. Advancing technology allows a greater number of people, including those living in remote areas to access information online using computers and smartphones. Digital solutions such as videos are excellent resources for education via multimedia for the teaching-learning process, facilitating the dissemination of diverse information^(11)^. Therefore, given the limited dissemination of knowledge about POP conservative management using vaginal pessary and its benefits for women facing this problem, proposing strategic and effective ways to accept pessary use is essential.

This study arises from a clear purpose of bringing together SDG 3 (Health and well-being) and SDG 5 (Gender equality) and their relationship so that women living with POP can enjoy their full life potential with quality and autonomy. Considering the above, the study is justified for nursing practice by bringing technology aimed at the care of patients with POP, in addition to contributing to the dissemination of a device that can favor the achievement of goals: 3.7: by 2030, ensuring the universal access to sexual and reproductive healthcare services, including family planning, information and education as well as the integration of reproductive health into national strategies and programs; 5.6: ensuring universal access to sexual and reproductive health and reproductive rights; and 5.b: increasing the use of basic technologies, in particular Information and Communication Technologies (ICTs), to promote women’s empowerment^(10)^.

## OBJECTIVES

To create and assess an educational video to promote adherence of women with POP to vaginal pessary use.

## METHODS

### Ethical aspects

The data analyzed in this study come from a master’s thesis. The present study complied with all ethical aspects concerning the development of research with human beings, as described in Resolution 466/2012 of the Brazilian National Health Council. The *Universidade Federal do Ceará* Research Ethics Committee approved this study. In compliance with relevant legislation recommendations, all people who participated in the research signed the Informed Consent Form (ICF).

### Study design, period and location

This is a methodological study, with a longitudinal design and quantitative analysis^(12)^. The process of creating the video, validity by experts and assessment by the target audience lasted 24 months. Data collection with judges and the target audience took place between January 2014 and November 2015. The research was carried out in the *Hospital Geral de Fortaleza* (HGF) urogynecology service. The institution was chosen because it provides specialized assistance to women with POP. The service uses pessaries with a standardized care protocol for insertion and monitoring, having been a pioneer in the state of Ceará.

### Population; inclusion and exclusion criteria

According to the literature, there is still no consensus on the number of judges needed to assess content. To define the sample size of judges and the target population, a formula was adopted that considers the final proportion of subjects in relation to a given dichotomous variable and the maximum acceptable difference in this proportion. To this end, the formula n=Zα2.P.(1-P)/d2 was used, where Zα refers to the level of confidence (95% was agreed), P is the proportion of individuals who agree with the relevance of video concepts/scenes and d is the difference in proportion considered acceptable^(13)^.

The final calculation was determined by n=1.962.0.85.0.15/0.152. A value of 22 was obtained, however, when adding a value of 10% for possible losses, the final value was reached of 24 participants, for educational video assessment (12 from the health area and 12 from the communication area), and 24 women from the HGF urogynecology service, for assessment by the target population.

Judges were divided into two areas: health (professionals with experience with pelvic floor dysfunction (PFD)) and communication. To define an expert and to identify and recruit evaluators, Jasper’s criteria were adopted^(14)^ (1994). Judges were selected using network or snowball sampling, which is used to locate samples that are difficult to find in any other way. With regard to the target audience, women were selected randomly according to demand, therefore, being a consecutive sample^(12)^.

Judges who met the minimum characteristics were invited to participate in the study via email. An electronic form was developed using Google Forms® to facilitate the participation of judges from other Brazilian states and regions. Judges’ forms were divided into three parts: 1) ICF; 2) judge identification data (graduation, length of training, practice time in the area, participation in groups/research projects and scientific production); and 3) instructions for filling out the instrument and assessment items from the script and video.

### Study protocol

Data collection as well as the instruments used varied depending on the process of educational video construction and assessment. In the development phase, the researchers monitored pessary consultations carried out at HGF for 12 months in order to identify the profile of the target population and establish video content and language. Construction followed three stages: pre-production (synopsis, argument, script, storyboard); production; and post-production^(15)^. In the pre-production and production phases, script and educational video content, technique and appearance were assessed.

**Figure 1:**
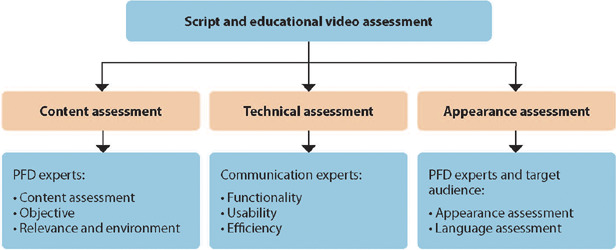
Educational script and video assessment, Fortaleza, Ceará, Brazil PAD – pelvic floor dysfunction.

First, the script was produced, content was assessed and, subsequently, the video was created. Script and video were assessed by health judges specializing in PFD regarding content (structure and presentation strategies, video objective, relevance and environment) and by communication judges regarding technique (video functionality, usability and efficiency) (Figure 1). To this end, an assessment instrument composed of 10 domains was used: 1. concept of the idea (promotion of adherence and follow-up to the use of pessaries for women with POP); 2. objectives (purposes, goals or ends that they want to achieve through the educational video); 3. dramatic construction (opening, conflict, development, climax and ending); 4. rhythm (evolution of dramatic moments and scene types); 5. characters (motivation, credibility and interaction); 6. dramatic potential; 7. dialogues (dramatic time); 8. visual style (aesthetics); 9. referent audience; and 10. relevance (characteristics that assess the degree of significance of images and scenes). Answers to questions are presented as excellent, very good, good, fair, lower fair and poor^(16)^. It should be noted that the aforementioned script and video assessment instruments contained space for comments. Appearance and language assessment was carried out by health judges specializing in PFD and by women (target audience) (Figure 1).

The target audience was consulted in order to assess educational video appearance and was made up of women with POP who use a pessary, identified at the HGF urogynecology service. To this end, an assessment instrument produced by the author was used, consisting of patient characterization and instructions for evaluative items from the video, covering five domains: 1. objectives; 2. organization; 3. language; 4. Appearance; and 5. motivation. Answers to questions are presented as yes, no and in part. Inclusion criteria for the target audience were women with POP who use a pessary followed at the HGF urogynecology service. Discontinuation criteria were women’s withdrawal from participating in the research after data collection began. Exclusion criteria were presenting a compromised physical or mental health condition that makes video assessment unfeasible.

### Analysis of results, and statistics

The records made by participants, nurses and students were stored in a Microsoft Office Excel® spreadsheet, online version, for descriptive statistical analysis (absolute frequency, relative frequency, mean and standard deviation). Content, technique and appearance assessment, carried out by expert judges, used analysis based on the binomial test application for adequacy of adjustment, in addition to using the Content Validity Index (CVI) considering a CVI greater than 0.80 as appropriate^(17, 18)^. With regard to the target audience, items that obtained a minimum level of agreement of 75% in positive responses were considered assessed for appearance^(19)^.

## RESULTS

**Figure 2:**
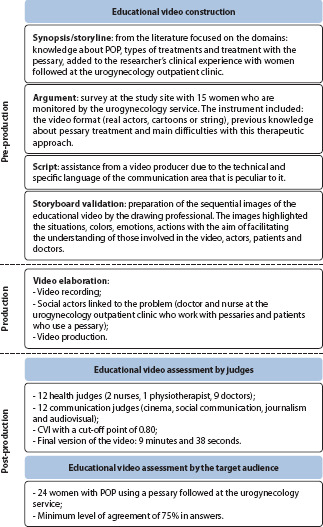
Flowchart of the process of educational video construction and assessment, Fortaleza, Ceará, Brazil POP – pelvic organ prolapse; CVI – Content Validity Index.

Figure 2 presents the process of educational video construction, validity and assessment by the target audience on promoting the adherence of women with POP to vaginal pessary use.

### Educational video construction

For synopsis or storyline development, the idea for this educational video resulted from the researcher’s empirical experience during the monitoring of patients using a pessary in a urogynecology outpatient clinic. It was observed that most women referred for their first pessary consultation were unaware of this therapeutic approach, often refusing to start treatment. Furthermore, when carrying out a broad search in national and international literature, the existence of educational technologies regarding vaginal pessary that would assist in the process of adherence and follow-up of treatment was not verified. Therefore, the following synopsis or storyline was prepared: “The video portrays situations through testimonies in which women demonstrate in their daily lives how they can use vaginal pessaries to correct the symptoms of pelvic organ prolapse, even in simpler socioeconomic contexts. Experiences lived by women and confirmed by healthcare professionals’ guidance will be able to improve the self-efficacy of these women in using vaginal pessary”.

Regarding the argument, this was carried out after pessary consultations, through a survey with 15 women who are monitored by the HGF urogynecology service to identify the format of the educational video that would be preferred by this audience. In the survey, questions were asked about video format (real actors, cartoons or *cordel* (booklet form of literature with woodprints or drawings recounting moral fables or hero-type stories, often sold in markets or on the street in northeastern Brazil)), previous knowledge about treatment and main difficulties with treatment. It is noteworthy that all women who were using pessaries in this service participated at the time of the survey. The sample was based on convenience, totaling 15 women. As for the educational video format, most women mentioned that they preferred to watch a video that was filmed with real people (N=13; 77.8%) rather than cartoons (N=1; 22.2%) or using *cordel* literature (N=1; 22.2%).

The video script was prepared based on the researchers’ experiences, with only one version being drafted before reaching the final version (second version). The initial version of the script was 15 minutes long. The scenes took place in a public park in the city of Fortaleza, at home and at the hospital where the study was carried out. In this version, the video was recorded with a nurse who participated in the initial scenes, contextualizing the topic, four patients undergoing treatment with a pessary and with the doctor from the HGF urogynecology service. In the second version of the script, some scenes were added by the scriptwriter collaborator, content and technical judges and the target audience; thus, the script went to ten minutes and 13 seconds. The educational video produced aims to share with women the signs and symptoms of POP as well as ways to treat this condition, emphasizing the conservative therapeutic approach using a pessary.

**Figure 3:**
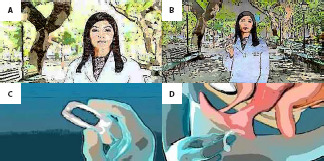
Excerpts from the storyboard presenting: A. Nurse and presenter reporting the concept of pelvic organ prolapse and its symptoms; B. Nurse providing guidance on the types of treatments for pelvic organ prolapse; C. Pessary; D. Pessary insertion, Fortaleza, Ceará, Brazil

To develop the storyboard, we had the help of a drawing professional, who created the sequential images for the educational video. It is possible to observe that the images sought to highlight the situations, colors, emotions and actions through the storyboard, with the aim of facilitating the understanding of those involved in the video, actors, patients and doctors about what was expected to be transmitted through this educational technology (Figure 3). Only the video producer, the researcher and the nurse who made the video initial presentation had access to the storyboard before filming, and it was used during rehearsals. The intention of not presenting the storyboard in advance to the other members of the video took place so that they would feel comfortable expressing their experiences in a truthful and natural way, as they were not actors.

The first version of the script (15 minutes) was submitted to the assessment process by content judges. A total of 12 judges from respected institutions in the scientific scene participated in this stage (*Universidade de São Paulo* (USP), *Universidade Federal do Ceará* (UFC), *Pontifícia Universidade Católica* (PUC), *Universidade Federal de São Paulo* (UNIFESP)), located in different Brazilian geographic regions (south, southeast and northeast). It should be noted that all content judges’suggestions were accepted so that, when reviewing the script, in order to reach the second version (final version), language became more simplified, with shorter sentences, seeking the help of a Portuguese proofreader. The title was changed from “*Pessários vaginais, você também pode usar!” to “Vamos testar o pessário?”.*

The video production stage included social actors, i.e., people who are linked to the problem. In this regard, the doctor who works directly in recommending pessary treatment, a nurse also involved in the urogynecology outpatient clinic and patients who use the vaginal pessary were invited. The choice of real characters was made to provide greater veracity to the scenes and to suit the characteristics of video characters.

For recording, we relied on the work of a professional specialized in video production, who had the necessary professional technical equipment, such as professional cameras, tripods, lighting and appropriate screens. Video recording lasted two days. First, the scenes in the park were recorded and the testimonies at the end of the video were recorded. On the second day, the focus was on recording images of the doctor and the main character (Dona Elzi) and her family, which took place in a urogynecology outpatient clinic, in the patient’s home and its surroundings.

### Educational video assessment by experts

Concerning expert characterization, it was found that the mean age of the judges participating in this study was 40.5 years, with a standard deviation of ± 8.6 years, median of 42.5 years, ranging from 27 to 52 years. Judges’ mean length of training was 16.2 years, with a standard deviation of ± 9.3 years, median of 13 years, ranging from three to 30 years of training. Five judges are male, and regarding training, two are nurses, one a physiotherapist, and nine doctors specializing in PFD.

In addition to assessment by health judges, the video was simultaneously assessed by 12 judges considered experts in communication (technical judges). We sought to contact judges from different Brazilian regions, with a representative sample of judges from the Southeast, Northeast and Central-West of Brazil. The mean age of the technical judges participating in this study was 34 years, with a standard deviation of ± 8.6 years old, median of 30 years old, ranging from 23 to 50 years. All judges were male. They worked in the areas of cinema, social communication, journalism and audiovisual, with specializations in cinema and scriptwriting. Length of training was on average 9.4 years, with a standard deviation of ± 3.3 years. The median was 8.5 years, ranging from six to 19 years.

**Figure 4:**
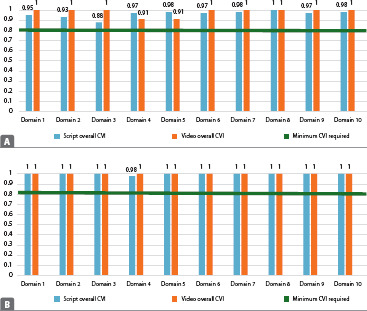
(A) Level of agreement among health judges based on evaluative aspects of content (n=12); (B) Level of agreement among communication judges based on evaluative aspects of content (n=12), Fortaleza, Ceará, Brazil CVI – Content Validity Index.

Concerning video content assessment, 11 judges (91.6%) approved the video without modifications and one approved it with modifications (8.3%). All suggestions were incorporated into the video. Regarding assessment by technical judges, everyone approved the video, without modifications, and no suggestions or modifications to the product were made. The educational video proved to be assessed material from the point of view of appearance and content, as it presented a satisfactory overall CVI (0.99) based on assessment by judges and an excellent level of agreement among judges (91.1% to 100%) (Figure 4).

### Educational video assessment by the target audience

The target population also carried out the assessment to check the appearance and language of the scenes that would make up the video. A total of 24 women with POP participated in the urogynecology service, with a sociodemographic profile similar to that of the target population of the video.

Participants’ ages ranged from 45 to 98 years (M:67.5±11.6). Sociodemographic characteristics demonstrate that most women have low education, income of up to three minimum wages. In relation to gynecological-obstetric and clinical characteristics, the entire sample was in menopause (100%/M: 68.7±11.06 years), with the majority being multiparous (87.5%), with an average of 6.5 (±4.3) pregnancies per woman. The most frequent route of delivery was vaginal (91.7%/M:5.58±4.3), and the largest newborn’s weight varied between 3.040 g and 5.050 g (M = 3.799 g (±1.423).

**Figure 5:**
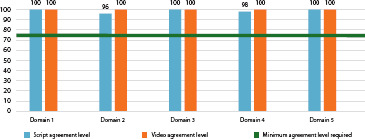
Level of agreement among the target audience (n=24), Fortaleza, Ceará, Brazil

Most patients sought care with the main complaint being the feeling of a ball in the vagina (96%), whereas 40% reported associated urinary complaints, such as urinary frequency (12%) or some type of incontinence (28%). On physical examination using the pelvic organ prolapse quantification system (POP-Q), 20% of patients had stage II prolapse, 44% stage III, and 36% stage IV. All women had defects of the anterior vaginal wall (100%). Assessment by the target audience obtained a high level of agreement (96% to 100%) (Figure 5).

Thus, 23 women (95.8%) approved the video without modifications and only one (4.2%) approved it with modifications. Suggested modifications were to add some scenes. It should be noted that the suggestions and modifications to be made were verified. To take into account judges’ and target audience’s suggestions, new scenes were added, requiring an additional day of recording. Given the suggestions and contributions arising from the assessment process, the educational video underwent modifications, adjustments and additions in order to make it more effective. Even though it achieved a favorable CVI, the video was revised and, even with the addition of some scenes, the final edit of the video was nine minutes and 38 seconds long, and is freely available on YouTube®, with 63 thousand views.

The educational video “*Vamos Testar o Pessário?”* can be accessed through the link: https://youtu.be/xPMX4n4VfMw. The UFCTV interview about the media can be accessed through the link: https://youtu.be/qQpYhbOZf_4.

## DISCUSSION

The term “digital health” currently appears to highlight a relevant field of practice for the use of routine and innovative forms of ICTs to meet health needs and improve care provision and management^(20)^. Following the trend of accelerated evolution of the digital era, ICTs are increasingly being used in health actions and strategies. These technologies favor the dissemination of information on health indicators, collection of epidemiological data, intersectoral communication, health promotion, health education^(21)^ and public health result improvement^(22)^.

Furthermore, 100 videos posted on YouTube® about POP were analyzed and measured for the quality of information. The search for videos occurred using the search term “pelvic organ prolapse”. Considering the DISCERN questionnaire metrics and the patient education materials assessment tool, only 10% (29 videos) were considered to be of high quality in terms of the content provided. Topics discussed in the videos were treatment options, benefits, risks, shared decision-making, and quality of life issues for patients with POP^(23)^. Pessary was discreetly mentioned in one of the results tables as a form of treatment, but no emphasis was given to the device.

Although a range of digital resources related to health are available to the population, mostly through mobile health applications (mHealth), It is observed that few scientific and technological developments have been produced in recent years to meet the demand to provide concise and relevant information about the pessary, and this should be considered among the first choice strategies for POP treatment^(24)^. Another survey developed across three social media platforms (YouTube®, Instagram® and Pinterest®) recorded only 21.0% (64/226) of posts related to pessary use^(22)^. The validity of using a pessary as a form of treatment is deliberately reinforced in specialized literature^(22, 24)^, but this treatment measure is little explored on social networks and media.

It is observed that, depending on the type of mHealth, there is a variation regarding the purpose disseminated on issues related to POP. For instance, on Instagram® and Pinterest®, posts focus on health and wellness, educating women on pelvic floor muscle training. On YouTube®, the main topic in focus was the surgical procedure to correct the dysfunction. It is worth mentioning that, in the referenced study, the content of videos on YouTube® had the worst comprehensibility, the lowest quality and the highest level of misinformation^(22)^, since they are presented by people without scientific expertise on the subject and who do not consider the limitations of the public that could learn from the content conveyed^(25)^.

Study participant characteristics demonstrate a wide variation in age, low education and low socioeconomic level. The profile of participants confirms that technologies aimed at this audience must have accessible language to meet all educational levels. The risk factors for POP found in participants were vaginal birth, multiparity and menopause, supporting the literature. In addition to these factors, others listed are age, birth weight, Body Mass Index^(26, 27, 28, 29)^, perineal ruptures and duration of the first labor >24 hours^(29)^. Knowing the characteristics of the population for which the educational technology is intended is of great value in adapting the material to the context of the target audience. It is at this moment that the researcher will be able to define the characteristics and type of technology that best suits each reality^(25)^, considering the diversity of means of disseminating information, such as social networks.

It is essential that women who seek content about POP via the internet are instructed by their health providers regarding the adoption of criteria for evidence-based accreditation of content available in the media. Karsalia and Malik^(30)^ (2022) reaffirm the seriousness of assessing the quality of information available in mHealth, considering that there is no eligibility criteria defined for the authors of posts and/or dissemination of content, with the only precept being access to the internet. Based on this, the educational video must be considered a reputable and valid tool, since, for its dissemination in the media, a dense and careful assessment is essential throughout the design process, as is the methodological path of this study: *Vamos Testar o Pessário?* Studies published with a similar methodology for creating educational videos corroborate this statement^(31, 32, 33)^.

A pessary as a method for treating POP should be rethought as a primary strategy, given that it is a proven effective measure and can also be adopted to minimize the burden on public healthcare services when compared to high-cost actions such as corrective surgery^(34)^. Based on the vision of a globalized and connected world, the educational video presented here, in addition to providing clarifications, instructions and entertainment, can serve as a driver to achieve SDGs 3.7, 5.6 and 5.b^(10)^, contributing to improving quality of life of women with POP.

### Study limitations

As limitations of this investigation, we can mention the absence of testimonies from sexually active women who use pessary due to the fact that the video was not recorded with actors, but rather with women who experience the problem, and at the time of recording there was no patient who fit this characteristic. Another limitation was the absence of scenes showing device insertion by patients themselves, but this was a behavior adopted by the researcher as a way of preserving study participant privacy.

### Contributions to nursing, health or public policy

The findings can expand the scope of knowledge, aiming to improve adherence to conservative treatment with vaginal pessary use for women with POP and, consequently, their quality of life. The data from this research can guide health and continuing education policies that qualify not only women who are recommended to use a pessary, but also healthcare professionals. Moreover, the video “*Vamos Testar o Pessár¡o?”* is a credible strategy for achieving SDGs 3.7, 5.6 and 5.b, as it is a media resource with an emphasis on encouraging well-being, empowerment and use of ICTs by women living with POP.

## CONCLUSIONS

The educational video in this study was the first to be developed within the topic in the Brazilian scenario and was positively assessed by health and communication professionals in terms of appearance and content, presenting an overall CVI (0.99) and an excellent level of agreement among judges (91.1% to 100%), and by representatives of the target audience (96% to 100%), and should therefore be considered in the context of educational activities as an instrument capable of promoting adherence to conservative practices for pessary treatment in women indicated for this therapeutic approach.

Using this material with women with POP during hospitalization in specialized services will facilitate healthcare professionals’ practice, as it constitutes a dynamic technology capable of fostering dialogue between professionals and women, facilitating knowledge acquisition for them, memorization of care related to treatment with pessary, providing women’s empowerment as well as becoming a means of standardizing the guidance given by professionals. The video is an excellent tool for promoting treatment, making it possible to apply it not only to the population, but also to healthcare professionals, helping Brazil achieve the goals agreed in the UN 2030 Agenda.
