# Supplementary material for: Educational video for adherence to vaginal pessary in pelvic organ prolapse treatment
Source: Rev Bras Enferm. 2024 Sep 30;77(Suppl 2):e20230515. doi: 10.1590/0034-7167-2023-0515 (PMC11441755; doi:10.1590/0034-7167-2023-0515)
Supplement: Translation

# Translation: Vídeo educativo para adesão ao pessário vaginal no tratamento do prolapso de órgãos pélvicos

**DOI:** 10.1590/0034-7167-2023-0515pt

**Authors:** Karine de Castro Bezerra, Maria Luziene de Sousa Gomes, Roger Rodrigues da Silva, Dayana Maia Saboia, José Ananias Vasconcelos, Camila Moreira Teixeira Vasconcelos, Mônica Oliveira Batista Oriá

**Affiliations:** I Universidade Federal do Ceará. Fortaleza, Ceará, Brasil.; II Universidade Federal do Maranhão. São Luís, Maranhão, Brasil.; III Universidade Federal do Ceará, Complexo Hospitalar, Empresa Brasileira de Serviços Hospitalares. Fortaleza, Ceará, Brasil.

**Keywords:** Comunicação em Saúde, Filme e Vídeo Educativo, Prolapso de Órgão Pélvico, Pessários, Cooperação e Adesão ao Tratamento

## Abstract

**Objetivos:**

construir e avaliar um vídeo educativo para promoção da adesão de mulheres com prolapso de órgãos pélvicos ao uso do pessário vaginal.

**Métodos:**

trata-se de estudo metodológico, com delineamento longitudinal e análise quantitativa. Foram percorridas as etapas de pré-produção (sinopse, argumento, roteiro, *storyboard),* produção e pós-produção. As avaliações de conteúdo e técnica foram realizadas por juízes da área da saúde e comunicação, respectivamente, e avaliação de aparência pelo público-alvo.

**Resultados:**

o vídeo foi o primeiro a ser desenvolvido sobre a temática no cenário nacional, considerado avaliado do ponto de vista de aparência e conteúdo, apresentando Índice de Validade de Conteúdo global de 0,99 e nível de concordância entre os juízes de 91,1% a 100%. A avaliação pelo público-alvo alcançou um percentual de 96% a 100%.

**Conclusões:**

o vídeo educativo é um instrumento capaz de promover a adesão ao pessário em mulheres com indicação para essa abordagem terapêutica.

## INTRODUÇÃO

O prolapso de órgãos pélvicos (POP) é o deslocamento para baixo do útero e/ou compartimentos da parede vaginal e seus órgãos associados (bexiga, reto e intestino)^(1)^. O POP traz implicações em vários domínios da qualidade de vida, contribuindo para alterações nas condições de saúde física, funções cognitivas, satisfação sexual, nas atividades do cotidiano, no bem-estar emocional, na vida familiar e social, ocasionando problemas sexuais, isolamento social, baixa autoestima e depressão^(2, 3)^. A prevalência de POP varia muito entre os estudos em cerca de 3% a 50%^(4)^. Mulheres com POP apresentam uma variedade de sintomas urinários, intestinais e sexuais que podem comprometer significativamente o bem-estar psicológico, emocional, bem como sua qualidade de vida^(5)^.

As opções de tratamento do POP incluem medidas conservadoras com exercícios para os músculos do assoalho pélvico e uso de pessário vaginal, além de medidas mais intervencionistas, como a cirurgia^(6, 7)^. O pessário vaginal é um dispositivo inserido na vagina para fornecer suporte estrutural a um ou mais dos compartimentos vaginais descendentes. Há uma grande variedade de pessários disponíveis no mercado. São dispositivos intravaginais, geralmente de silicone, que sustentam os órgãos pélvicos e colocam esses na posição anatomicamente correta. Além disso, permitem o controle sintomático com o benefício adicional de adiar ou evitar a intervenção cirúrgica^(1, 8)^.

Apesar dos muitos benefícios que o pessário oferece, a escolha e continuidade desse tratamento pela mulher é complexa e passa por esferas relacionadas ao conhecimento, à motivação e à confiança^(9)^. Intervenções educativas podem maximizar a aceitação de mulheres com POP ao uso do pessário, além de melhorar a assistência à saúde em relação à adesão e ao acompanhamento das pacientes que fazem uso do dispositivo.

Sob essa perspectiva, esse tópico se insere nos objetivos 3 (assegurar uma vida saudável e promover o bem-estar para todos, em todas as idades) e 5 (alcançar a igualdade de gênero e empoderar todas as mulheres e meninas) elencados entre os Objetivos de Desenvolvimento Sustentável (ODS) Agenda 2030 da Organização das Nações Unidas (ONU)^(10)^.

Um método de intervenção educativa para melhorar a literacia sobre o manejo conservador do POP por meio do pessário vaginal é o meio digital. O avanço da tecnologia permite que um maior número de pessoas, incluindo aquelas que vivem em áreas remotas, acesse informações *online* usando computadores e *smartphones.* As soluções digitais como vídeos são excelentes recursos para educação via multimídia para o processo de ensino-aprendizagem, facilitando a divulgação de informações diversas^(11)^. Dessa forma, diante da pouca difusão de conhecimento sobre o manejo conservador do POP por meio do pessário vaginal e seus benefícios para mulheres que enfrentam essa problemática, propor formas estratégicas e eficazes para aceitação do uso do pessário é fundamental.

Este estudo surge a partir de um claro propósito de aproximação entre o ODS 3 (Saúde e bem-estar) e o ODS 5 (Igualdade de gênero) e sua relação para que as mulheres que convivem com POP desfrutem de todo o seu potencial de vida com qualidade e autonomia. Diante do exposto, o estudo justifica-se para a prática da enfermagem por trazer uma tecnologia voltada para o cuidado de pacientes com POP, além de contribuir para a divulgação de um dispositivo que pode favorecer o alcance das metas: 3.7: até 2030, assegurar o acesso universal aos serviços de saúde sexual e reprodutiva, incluindo o planejamento familiar, informação e educação, bem como a integração da saúde reprodutiva em estratégias e programas nacionais; 5.6: assegurar o acesso universal à saúde sexual e reprodutiva e os direitos reprodutivos; e 5.b: aumentar o uso de tecnologias de base, em particular as Tecnologias de Informação e Comunicação (TICs), para promover o empoderamento das mulheres^(10)^.

## OBJETIVOS

Construir e avaliar um vídeo educativo para a promoção da adesão de mulheres com POP ao uso do pessário vaginal.

## MÉTODOS

### Aspectos éticos

Os dados analisados neste estudo são oriundos de uma dissertação de mestrado. O presente estudo cumpriu todos os aspectos éticos concernentes ao desenvolvimento de pesquisas com seres humanos, conforme descrito na Resolução nº 466/2012 do Conselho Nacional de Saúde. O Comitê de Ética em Pesquisa da Universidade Federal do Ceará aprovou este estudo. Em atenção às recomendações da legislação pertinente, todas as pessoas que participaram da pesquisa assinaram o Termo de Consentimento Livre e Esclarecido (TCLE).

### Desenho, período e local do estudo

Trata-se de estudo metodológico, com delineamento longitudinal e análise quantitativa^(12)^. O processo de construção do vídeo, validação por peritos e avaliação pelo público-alvo durou 24 meses. A coleta de dados com juízes e o público-alvo se deu entre o período de janeiro de 2014 a novembro de 2015. A pesquisa foi desenvolvida no serviço de uroginecologia do Hospital Geral de Fortaleza (HGF). A escolha da instituição se deu por prestar assistência especializada às mulheres com POP. O serviço utiliza pessários com protocolo de atendimento padronizado de inserção e acompanhamento, tendo sido pioneiro no estado do Ceará.

### População; critérios de inclusão e exclusão

Segundo a literatura, ainda não há um consenso sobre o número de juízes necessários para a avaliação de conteúdo. Para definição do tamanho amostral dos juízes e da população alvo, adotou-se a fórmula que considera a proporção final dos sujeitos no tocante à determinada variável dicotômica e a diferença máxima aceitável dessa proporção. Para tanto, utilizou-se a fórmula n=Zα2.P.(1-P)/d2, em que Zα refere-se ao nível de confiança (convencionou-se 95%), P é a proporção de indivíduos que concordam com a pertinência dos conceitos/cenas do vídeo e d é a diferença de proporção considerada aceitável^(13)^.

O cálculo final foi determinado por n=1,962.0,85.0,15/0,152. Obteve-se um valor de 22, contudo, ao acrescentar um valor de 10% para possíveis perdas, chegou ao valor final de 24 participantes, para avaliação do vídeo educativo (12 da área da saúde e 12 da área da comunicação) e 24 mulheres do serviço de uroginecologia do HGF, para avaliação pela população-alvo.

Os juízes foram divididos em duas áreas: saúde (profissionais com experiência com disfunção do assoalho pélvico (DAP)) e comunicação. Para definição de *expert* e para identificar e recrutar os avaliadores, foram adotados os critérios de Jasper^(14)^ (1994). A seleção dos juízes se deu por meio da amostragem de rede ou bola de neve, a qual é utilizada para localizar amostras difíceis de serem encontradas de outra maneira. No que se refere ao público-alvo, as mulheres foram selecionadas de forma aleatória conforme a demanda, sendo, portanto, uma amostragem consecutiva^(12)^.

Os juízes que atingiram as características mínimas foram convidados a participar do estudo via e-mail. Foi desenvolvido um formulário eletrônico por meio do aplicativo *Google Forms*®, para facilitar a participação de juízes de outros estados e regiões brasileiras. Os formulários dos juízes foram divididos em três partes: 1) TCLE; 2) dados de identificação do juiz (graduação, tempo de formação, tempo de prática na área, participação em grupos/projetos de pesquisa e produção científica); e 3) instruções para preenchimento do instrumento e itens avaliativos do roteiro e do vídeo.

### Protocolo do estudo

A coleta de dados, assim como os instrumentos utilizados, variou a depender do processo de construção e avaliação do vídeo educativo. Na fase de desenvolvimento, os pesquisadores acompanharam, durante 12 meses, as consultas de pessário realizadas no HGF, a fim de identificar o perfil da população-alvo e estabelecer o conteúdo e a linguagem do vídeo. A construção seguiu três etapas: pré-produção (sinopse, argumento, roteiro, *storyboard*); produção; e pós-produção^(15)^. Nas fases de pré-produção e produção, foram avaliados conteúdo, técnica e aparência do roteiro e do vídeo educativo.

Primeiro foi realizada a produção do roteiro, avaliação de conteúdo e, posteriormente, a construção do vídeo. O roteiro e o vídeo foram avaliados pelos juízes da área da saúde especialistas em DAP quanto ao conteúdo (estrutura e estratégias de apresentação, objetivo, relevância e ambiente do vídeo) e pelos juízes da comunicação quanto à técnica (funcionalidade, usabilidade e eficiência do vídeo) (Figura 1). Para tanto, foi utilizado um instrumento de avaliação composto por dez domínios: 1. conceito da ideia (promoção da adesão e seguimento ao uso do pessário da mulher com POP); 2. objetivos (propósitos, metas ou fins que se deseja atingir por meio do vídeo educativo); 3. construção dramática (abertura, conflito, desenvolvimento, clímax e final); 4. ritmo (evolução dos momentos dramáticos e tipos de cena); 5. personagens (motivação, credibilidade e interação); 6. potencial dramático; 7. diálogos (tempo dramático); 8. estilo visual (estética); 9. público referente; e 10. relevância (características que avaliam o grau de significação das imagens e cenas). As respostas às perguntas são apresentadas como excelente, muito bom, bom, regular, regular inferior e pobre^(16)^. Ressalta-se que os referidos instrumentos de avaliação do roteiro e do vídeo continham espaço para comentários. Já a avaliação da aparência e linguagem foi feita pelos juízes da área da saúde especialistas em DAP e pelas mulheres (público-alvo) (Figura 1).

**Figura 1:** Avaliação do roteiro e do vídeo do educativo, Fortaleza, Ceará, Brasil DAP – disfunção do assoalho pélvico.

O público-alvo foi consultado a fim de se realizar a avaliação da aparência do vídeo educativo, e foi constituído por mulheres com POP que fazem uso do pessário, captadas no serviço de uroginecologia do HGF. Para tanto, foi utilizado um instrumento de avaliação produzido pela autora, composto pela caracterização da paciente e as instruções de itens avaliativos do vídeo, contemplando cinco domínios: 1. objetivos; 2. organização; 3. linguagem; 4. Aparência; e 5. motivação. As respostas às perguntas são apresentadas como sim, não e em parte. Os critérios de inclusão para o público-alvo foram mulheres com POP que fazem uso do pessário acompanhadas no serviço de uroginecologia do HGF. Os critérios de descontinuidade foram desistência da mulher em participar da pesquisa após início da coleta de dados. Os critérios de exclusão foram apresentar estado de saúde físico ou mental comprometido, de modo a inviabilizar a avaliação do vídeo.

### Análise dos resultados e estatística

Os registros realizados pelos participantes, enfermeiras e estudantes foram armazenados em uma planilha no *Microsoft Office Excel*®, versão *online,* para análise estatística descritiva (frequência absoluta, frequência relativa, média e desvio padrão). A avaliação de conteúdo, técnica e aparência, realizada por juízes especialistas, utilizou a análise baseada na aplicação do teste binomial para adequação do ajuste, além da utilização do Índice de Validade de Conteúdo (IVC) considerando um IVC maior que 0,80 como apropriado^(17, 18)^. No que se refere ao público-alvo, os itens que obtiveram um nível mínimo de concordância de 75% em respostas positivas foram considerados avaliados quanto à aparência^(19)^.

## RESULTADOS

O processo de construção, validação e avaliação pelo público-alvo do vídeo educativo sobre promoção da adesão de mulheres com POP ao uso do pessário vaginal é apresentado na Figura 2.

**Figura 2:** Fluxograma do processo de construção e avaliação pelo público-alvo do vídeo educativo, Fortaleza, Ceará, Brasil POP – prolapso de órgãos pélvicos; IVC – Índice de Validade de Conteúdo.

### Construção do vídeo educativo

Para o desenvolvimento da sinopse ou *storyline,* a ideia do presente vídeo educativo resultou da experiência empírica da pesquisadora durante o acompanhamento das pacientes que utilizam o pessário em um ambulatório de uroginecologia. Observou-se que grande parte das mulheres encaminhadas para a primeira consulta de pessário desconhecia essa abordagem terapêutica, recusando muitas vezes iniciar o tratamento. Além disso, ao realizar ampla busca na literatura nacional e internacional, não foi verificada a existência de tecnologias educativas acerca do pessário vaginal que auxiliassem no processo de adesão e seguimento do tratamento. Logo, elaborou-se a seguinte sinopse ou *storyline: “*O vídeo retrata situações através de depoimentos em que mulheres demonstram no seu cotidiano como conseguem usar o pessário vaginal para corrigir os sintomas do prolapso de órgãos pélvicos, mesmo em contextos socioeconómicos mais simples. Experiências vivenciadas pelas mulheres e ratificadas pelas orientações dos profissionais de saúde serão capazes de melhorar a autoeficácia dessas mulheres em utilizar o pessário vaginal”.

Com relação ao argumento, esse foi realizado após as consultas de pessário, mediante uma enquete com 15 mulheres que são acompanhadas pelo serviço de uroginecologia do HGF para a identificação do formato do vídeo educativo que seria preferido por esse público. Na enquete, foram questionados o formato do vídeo (atores reais, desenhos animados ou cordel), conhecimento anterior acerca do tratamento e principais dificuldades com o tratamento. Ressalta-se que participaram todas as mulheres que estavam utilizando pessário nesse serviço até a data da enquete. A amostra deu-se por conveniência totalizando em 15 mulheres. Com relação ao formato do vídeo educativo, a maioria das mulheres mencionou que preferia assistir a um vídeo que fosse filmado com pessoas reais (N=13; 77,8%) ao invés de desenhos animados (N=1; 22,2%) ou utilizando literatura de cordel (N=1; 22,2%).

O roteiro do vídeo foi elaborado a partir das experiências das pesquisadoras, tendo sido esboçada apenas uma versão antes que se chegasse à versão final (segunda versão). A versão inicial do roteiro constava 15 minutos. As cenas passavam-se em parque público no município de Fortaleza, em domicílio e no hospital no qual o estudo foi realizado. Nessa versão, o vídeo foi gravado com uma enfermeira que participou das cenas iniciais contextualizando a temática, quatro pacientes que fazem tratamento com pessário e com o próprio médico do serviço de uroginecologia do HGF. Na segunda versão do roteiro, foram acrescentadas algumas cenas pelo colaborador roteirista, pelos juízes de conteúdo e técnicos e pelo público-alvo; assim, o roteiro passou para dez minutos e 13 segundos. O vídeo educativo produzido tem o intuito de compartilhar com mulheres os sinais e sintomas do POP, bem como as formas de tratamento dessa condição, dando ênfase à abordagem terapêutica conservadora utilizando o pessário.

Para o desenvolvimento do *storyboard,* contou-se com o auxílio de um profissional da área de desenhos, o qual elaborou as imagens sequenciais do vídeo educativo. É possível observar que as imagens buscaram evidenciar as situações, cores, emoções e ações por meio do *storyboard,* com o intuito de facilitar a compreensão dos envolvidos no vídeo, atores, pacientes e médico acerca do que se esperava transmitir por meio dessa tecnologia educativa (Figura 3). Apenas o produtor do vídeo, a pesquisadora e a enfermeira que realizou a apresentação inicial do vídeo tiveram acesso ao *storyboard* antes da filmagem, sendo utilizado durante os ensaios. O intuito em não apresentar o *storyboard* previamente para os outros integrantes do vídeo se deu para que eles se sentissem à vontade para expressar suas experiências de forma verídica e natural, já que não eram atores.

**Figura 3:** Trechos do *storyboard* apresentando: A. Enfermeira e apresentadora relatando o conceito de prolapso de órgãos pélvicos e seus sintomas; B. Enfermeira orientando sobre os tipos de tratamentos para POP; C. Pessário; D. Inserção do Pessário, Fortaleza, Ceará, Brasil

A primeira versão do roteiro (15 minutos) foi submetida ao processo de avaliação por parte dos juízes de conteúdo. Participaram dessa etapa 12 juízes de instituições respeitadas no cenário científico (Universidade de São Paulo (USP), Universidade Federal do Ceará (UFC), Pontifícia Universidade Católica (PUC), Universidade Federal de São Paulo (UNIFESP)), localizadas em diferentes regiões geográficas brasileiras (sul, sudeste e nordeste). Ressalta-se que todas as sugestões dos juízes de conteúdo foram acatadas de forma que, na revisão do roteiro, para que se chegasse à segunda versão (versão final), a linguagem tornou-se mais simplificada, com frases mais curtas, buscando-se, para tanto, o auxílio de um revisor de português. O título foi modificado passando de “Pessários vaginais, você também pode usar!” para “Vamos testar o pessário?”

A etapa de produção do vídeo contou com atores sociais, ou seja, pessoas que estão ligadas à problemática. Nesse sentido, convidaram-se o médico que trabalha diretamente na indicação do tratamento com pessário, uma enfermeira também envolvida no ambulatório de uroginecologia e as pacientes que utilizam o pessário vaginal. A escolha de personagens reais se deu por propiciar maior veracidade às cenas e pela adequação às características dos personagens do vídeo.

Para gravação, contou-se com o trabalho de um profissional especialista na área de produção de vídeos, o qual dispunha do equipamento técnico profissional necessário, tais como cameras profissionais, tripés, iluminação e anteparos apropriados. A gravação do vídeo durou dois dias. Primeiro, procedeu-se à gravação das cenas realizadas no parque e aos depoimentos do final do vídeo. No segundo dia, enfocou-se na gravação das imagens do médico e da personagem principal (Dona Elzi) e seus familiares, que ocorreu em um ambulatório de uroginecologia, na casa da paciente e nos arredores de sua casa.

### Avaliação do vídeo educativo pelos especialistas

Quanto à caracterização dos peritos, obteve-se que a média da idade dos juízes participantes deste estudo foi de 40,5 anos, com desvio padrão de ± 8,6 anos, mediana de 42,5 anos, variando de 27 a 52 anos. O tempo médio de formação dos juízes foi de 16,2 anos, com desvio padrão de ± 9,3 anos, mediana de 13 anos, variando de três a 30 anos de formação. Cinco juízes são do sexo masculino, e quanto à formação, dois são enfermeiros, um, fisioterapeuta, e nove, médicos especialistas em DAP.

Além da avaliação por juízes da área da saúde, o vídeo foi avaliado simultaneamente por 12 juízes considerados *experts* na área da comunicação (juízes técnicos). Buscou-se contatar juízes de diferentes regiões brasileiras, sendo amostra de juízes representativa do Sudeste, Nordeste e Centro-Oeste brasileiro. A média de idade dos juízes técnicos participantes deste estudo foi de 34 anos, com desvio padrão de ± 8,6 anos, mediana de 30 anos, variando de 23 a 50 anos. Todos os juízes eram do sexo masculino. Atuavam nas áreas de cinema, comunicação social, jornalismo e audiovisual, com especializações em cinema e roteiro. O tempo de formação foi em média 9,4 anos, com desvio padrão de ± 3,3 anos. A mediana foi de 8,5 anos, variando de seis a 19 anos.

Acerca da avaliação do conteúdo do vídeo, 11 juízes (91,6%) aprovaram o vídeo sem modificações e um aprovou com modificações (8,3%). Todas as sugestões foram incorporadas ao vídeo. Em relação à avaliação pelos juízes técnicos, todos aprovaram o vídeo, sem modificações, não sendo realizadas sugestões ou modificações do produto. O vídeo educativo mostrou-se material avaliado do ponto de vista de aparência e conteúdo, visto que apresentou IVC global satisfatório (0,99) a partir da avaliação pelos juízes e nível de concordância excelente entre os juízes (91,1% a 100%) (Figura 4).

**Figura 4:** (A) Nível de concordância entre juízes da saúde por aspectos avaliativos de conteúdo (n=12); (B) Nível de concordância entre juízes da comunicação por aspectos avaliativos de conteúdo (n=12), Fortaleza, Ceará, Brasil IVC – Índice de Validade de Conteúdo.

### Avaliação do vídeo educativo pelo público-alvo

A população-alvo também realizou a avaliação para verificar a aparência e a linguagem das cenas que iriam compor o vídeo. Participaram 24 mulheres com POP acompanhadas no serviço de uroginecologia, com perfil sociodemográfico semelhante ao da população-alvo do vídeo.

A idade das participantes variou de 45 a 98 anos (M:67,5±11,6). As características sociodemográficas demonstram que grande parte das mulheres possui baixa escolaridade, renda de até três salários mínimos. Em relação às características gineco-obstétricas e clínicas, toda a amostra encontrava-se na menopausa (100%/M:68,7±11,06 anos), sendo a maioria multípara (87,5%), com média de 6,5 (±4,3) gestações por mulher. A via de parto mais frequente foi a vaginal (91,7%/M:5,58±4,3), e o peso do maior recém-nascido variou entre 3,040 g e 5,050 g (M = 3,799 g (±1,423).

A maioria das pacientes procurou atendimento apresentando como queixa principal a sensação de bola na vagina (96%), enquanto que 40% relataram queixas urinárias associadas, como polaciúria (12%) ou algum tipo de incontinência (28%). No exame físico pelo sistema de quantificação do prolapso de órgãos pélvicos (POP-Q), 20% das pacientes apresentavam prolapso estágio II, 44%, estágio III, e 36%, estágio IV. Todas as mulheres tinham defeitos da parede anterior da vagina (100%). A avaliação pelo público-alvo obteve elevado nível de concordância (96% a 100%) (Figura 5).

**Figura 5:** Nível de concordância entre o público-alvo (n=24), Fortaleza, Ceará, Brasil

Assim, 23 mulheres (95,8%) aprovaram o vídeo sem modificações e apenas uma (4,2%) aprovou com modificações. As modificações sugeridas foram para acrescentar algumas cenas. Ressalta-se que foram verificadas as sugestões e modificações a serem realizadas. Para contemplar as sugestões dos juízes e público-alvo, foram acrescentadas novas cenas, sendo necessário mais um dia de gravação. Diante das sugestões e contribuições oriundas do processo de avaliação, o vídeo educativo passou por modificações, ajustes e acréscimos a fim de torná-lo mais eficaz. Mesmo tendo alcançado IVC favorável, o vídeo foi revisado e, mesmo com o acréscimo de algumas cenas, a edição final do vídeo apresentou nove minutos e 38 segundos, e está disponível livremente na plataforma *YouTube*®, com 63 mil visualizações.

O vídeo educativo “Vamos Testar o Pessário?” pode ser acessado através do *link:* https://youtu.be/xPMX4n4VfMw. A entrevista da UFCTV sobre a mídia pode ser acessada através do link: https://youtu.be/qQpYhbOZf_4.

## DISCUSSÃO

O termo “saúde digital”, atualmente, surge evidenciando um campo de prática relevante para o emprego de formas rotineiras e inovadoras de TICs para atender às necessidades de saúde e melhorar a prestação e gestão de cuidados^(20)^. Acompanhando a tendência da acelerada evolução da era digital, as TICs estão sendo cada vez mais utilizadas nas ações e estratégias de saúde. Essas tecnologias favorecem para a disseminação de informações sobre indicadores em saúde, coleção de dados epidemiológicos, comunicação intersetorial, promoção da saúde, educação em saúde^(21)^ e melhoria dos resultados para a saúde pública^(22)^.

Além disso, 100 vídeos postados no *YouTube*® sobre POP foram analisados e mensurados quanto à qualidade das informações. A busca por vídeos ocorreu a partir do termo de pesquisa “prolapso de órgão pélvico”. Considerando as métricas do questionário DISCERN e da ferramenta de avaliação de materiais de educação do paciente, apenas 10% (29 vídeos) foram considerados de alta qualidade no que se refere ao conteúdo disposto. Os tópicos discutidos nos vídeos foram opções de tratamento, benefícios, riscos, tomada de decisão compartilhada e questões de qualidade de vida de pacientes com POP^(23)^. O pessário foi citado discretamente em uma das tabelas de resultados como forma de tratamento, mas nenhuma ênfase foi dada ao dispositivo.

Embora esteja disponível à população uma gama de recursos digitais que versam sobre saúde, majoritariamente através da modalidade de aplicativos móveis em saúde (*mHealth*), observa-se que poucos desenvolvimentos científicos tecnológicos foram produzidos nos últimos anos para atender à demanda de prestar informações concisas e relevantes sobre o pessário, sendo que esse deve ser considerado entre as estratégias de primeira escolha para o tratamento de POP^(24)^. Outra pesquisa desenvolvida através de três plataformas de mídias sociais (*YouTube*®, *Instagram*® e *Pinterest*®) registrou apenas 21,0% (64/226) de postagens relacionadas ao uso do pessário^(22)^. A validade em se utilizar o pessário como forma de tratamento é deliberadamente reforçada na literatura especializada^(22, 24)^, mas essa medida de tratamento é pouco explorada nas redes e mídias sociais.

Observa-se que, a depender do tipo de *mHealth,* existe uma variação quanto ao propósito difundido sobre assuntos relativos ao POP. Por exemplo, no *Instagram*® e *Pinterest*®, as postagens focam em saúde e bem-estar, instruindo as mulheres quanto ao treinamento da musculatura do assoalho pélvico. Já no *YouTube*®, o principal assunto em foco foi o procedimento cirúrgico para correção da disfunção. Vale mencionar que, no estudo referenciado, o conteúdo dos vídeos no *YouTube*® obteve a pior compreensibilidade, a qualidade mais baixa e o mais alto nível de desinformação^(22)^, uma vez que são apresentadas por pessoas sem *expertise* científica no assunto e que tampouco consideram as limitações do público que poderia instruir-se a partir do conteúdo veiculado^(25)^.

As características das participantes deste estudo demonstram uma ampla variação de idade, baixa escolaridade e baixo nível socioeconómico. O perfil das participantes ratifica que tecnologias voltadas para esse público devem ter linguagem acessível para atender a todos os níveis educacionais. Os fatores de risco para POP encontrados nas participantes foram parto vaginal, multiparidade e menopausa, corroborando a literatura. Além desses fatores, outros elencados são idade, peso ao nascer, Índice de Massa Corporal^(26, 27, 28, 29)^, rupturas perineais e duração do primeiro trabalho de parto >24 horas^(29)^. Conhecer as características da população a qual a tecnologia educativa destina-se é de grande valia para a adequação do material ao contexto do público-alvo. É nesse momento que o pesquisador poderá definir as características e o tipo de tecnologia que melhor condiz para cada realidade^(25)^, considerando a diversidade de meios difusores de informações, tais como as redes sociais.

É indispensável que as mulheres que buscam conteúdo sobre POP via *internet* estejam instruídas pelos seus provedores de saúde quanto à adoção de critérios para a creditação baseada em evidências do conteúdo disposto nas mídias. Karsalia e Malik^(30)^ (2022) reafirmam a seriedade em avaliar a qualidade das informações disponíveis nos *mHealth,* considerando que não há critério para elegibilidade definido sobre os autores de postagens e/ou divulgação de conteúdos, tendo como único preceito ter acesso à *internet.* A partir disso, o vídeo educativo deve ser considerado uma ferramenta conceituada e válida, pois, para a sua difusão nas mídias, é indispensável uma densa e criteriosa avaliação durante todo o processo de concepção, como o percurso metodológico do presente estudo: Vamos Testar o Pessário? Estudos publicados com metodologia análoga para criação de vídeos educativos corroboram essa declaração^(31, 32, 33)^.

O pessário como método para o tratamento de POP deve ser repensado como estratégia primária, haja vista que é uma medida comprovadamente eficaz, podendo ser adotada ainda para minimizar a oneração dos serviços públicos de saúde, quando comparados com ações de alto custo, como a cirurgia corretiva^(34)^. A partir da visão de um mundo globalizado e conectado, o vídeo educativo aqui apresentado, além de prestar esclarecimentos, instruções e entretenimento, pode servir como propulsor para alcançar os ODS 3.7, 5.6 e 5.b^(10)^, contribuindo para a melhoria da qualidade de vida de mulheres com POP.

### Limitações do estudo

Como limitações da presente investigação, pode-se mencionar a ausência de depoimentos de mulheres sexualmente ativas que utilizam o pessário pelo fato de o vídeo não ter sido gravado com atores, mas sim com mulheres que vivem a problemática, não havendo até a data das gravações nenhuma paciente que se enquadrasse nessa característica. Outra limitação foi a ausência de cenas que mostrassem a inserção do dispositivo pela própria paciente, porém essa foi uma conduta assumida pela pesquisadora como forma de preservar a intimidade das participantes do estudo.

### Contribuições para a área da enfermagem, saúde ou políticas públicas

Os achados podem ampliar o escopo de conhecimento, visando melhorar a adesão ao tratamento conservador com uso do pessário vaginal às mulheres com POP e, consequentemente, sua qualidade de vida. Os dados desta pesquisa podem orientar políticas de educação em saúde e permanente que qualifiquem não só as mulheres com indicação do uso do pessário, mas também os profissionais de saúde. Além disso, o vídeo “Vamos Testar o Pessário?” apresenta-se enquanto estratégia crível para o cumprimento dos ODS 3.7, 5.6 e 5.b, por ser um recurso midiático com ênfase ao estímulo do bem-estar, empoderamento e uso de TICs por mulheres que convivem com POP.

## CONCLUSÕES

O vídeo educativo deste estudo foi o primeiro a ser desenvolvido dentro do tema no cenário brasileiro e se mostrou avaliado positivamente por profissionais de saúde e da comunicação quanto à aparência e ao conteúdo, apresentando IVC global (0,99) e nível de concordância excelente entre os juízes (91,1% a 100%), e pelas representantes do público-alvo (96% a 100%), devendo, assim, ser considerado no contexto das atividades educativas como um instrumento capaz de promover a adesão a práticas conservadoras para o tratamento com o pessário em mulheres com indicação para essa abordagem terapêutica.

A utilização desse material com mulheres portadoras de POP na internação em serviços especializados facilitará a prática dos profissionais de saúde, uma vez que se constitui uma tecnologia dinâmica capaz de fomentar o diálogo entre profissionais e mulheres, facilitando a aquisição de conhecimentos por elas, a memorização de cuidados relacionados ao tratamento com o pessário, proporcionando o empoderamento da mulher, bem como se tornando um meio de padronizar as orientações dadas pelos profissionais. O vídeo é uma excelente ferramenta de divulgação do tratamento, sendo possível sua aplicação não só para a população, mas também para profissionais de saúde, contribuindo para que o Brasil alcance as metas pactuadas na Agenda 2030 da ONU.
